# Interleukin-27 as a Novel Biomarker for Early Cardiopulmonary Failure in Enterovirus 71-Infected Children with Central Nervous System Involvement

**DOI:** 10.1155/2016/4025167

**Published:** 2016-06-15

**Authors:** Mingyuan Huang, Wenjing Du, Jun Liu, Haiyang Zhang, Longbin Cao, Weiqing Yang, Hui Zhang, Zhiyong Wang, Pei Wei, Weiquan Wu, Zhulin Huang, Ying Fang, Qiling Lin, Xingwen Qin, Zhizhong Zhang, Keyuan Zhou, Jincheng Zeng

**Affiliations:** ^1^Department of Sanitary Analysis, Dongguan Key Laboratory of Environmental Medicine, School of Public Health, Guangdong Medical University, Dongguan 523808, China; ^2^Guangdong Provincial Key Laboratory of Medical Molecular Diagnostics, Dongguan Scientific Research Center, Guangdong Medical University, Dongguan 523808, China; ^3^Department of Integrative Medicine, Huashan Hospital, Fudan University, Shanghai 200040, China; ^4^Guangzhou Nansha Center for Disease Control and Prevention, Guangzhou 511458, China; ^5^Guangzhou Nansha Central Hospital, Guangzhou 511458, China

## Abstract

Enterovirus 71 (EV71) is a major pathogen for severe hand, foot, and mouth disease (HFMD), which leads to severe neurological complications and has high morbidity and mortality. Reliable biomarker for the prediction of deterioration in EV71-infected children with central nervous system (CNS) involvement may reduce the cardiopulmonary failure and mortality. Here, we found that serum IL-27 levels were significantly higher in stage III EV71-infected HFMD patients with early cardiopulmonary failure and strong correlation with CRP levels.* IL27p28* polymorphisms (rs153109, rs17855750, and rs181206) did not influence IL-27 production, and these three SNPs were not associated with EV71 infection risk and clinical stage. IL-27 can be used as an prediction indicator for early cardiopulmonary failure in EV71-infected children with CNS involvement.

## 1. Introduction

Hand, foot, and mouth disease (HFMD) is a viral infectious disease that commonly occurs in infants and young children (mostly ≤5 years of age). It is typically characterized by the sudden appearance of erythematous papulovesicular eruptions on the hand, feet, perioral area, knees, and buttocks and inside the oral cavity. Human coxsackievirus A16 (CA16) and enterovirus 71 (EV71) are the two major pathogens of HFMD. Generally, children with HFMD have mild symptoms that resolve spontaneously within 7–14 days without complications. However, some EV71-infected HFMD cases lead to severe neurological complications (aseptic meningitis, poliomyelitis-like paralysis, encephalitis, myocarditis, meningoencephalitis, and neonatal sepsis) and even death [1–3]. Besides the primary symptoms fever and erythema, our clinical data indicated some children with severe conditions have a body temperature >39°C and may appear with oral ulcer, being easily frightened, depression, abnormal breathing, being lethargic, being drowsy, vomiting frequently, and even convulse. However, to diagnose or predict the deterioration in EV71-infected children according to symptoms is limited. Most EV71-infected children with central nervous system (CNS) involvement have a good prognosis if they are diagnosed early and receive proper supportive treatment. Serum marker will be better than symptoms to predict the early cardiopulmonary failure in EV71-infected children with CNS involvement. Cardiopulmonary failure is the main cause of death. Therefore, reliable biomarker for the prediction of the early cardiopulmonary failure in EV71-infected children with CNS involvement may reduce the cardiopulmonary failure and mortality.

The mechanism of EV71 pathogenesis has been studied extensively, and the regulation of host immune responses is suspected to aggravate EV71-induced neurological symptoms [4–7]. Interleukin- (IL-) 27, a critical pleiotropic cytokine of the IL-12 family, is an important immunological regulator. It forms by the dimerization of Epstein-Barr virus-induced gene 3 (EBI3) and IL-27 p28. The latter, IL-27 p28, blocks the activity of some cytokines (e.g., IL-6, IL-11, and IL-27) [8]. It also promotes IL-10 production in virus-specific CD4^+^ T cells [9]. Our previous studies have shown that EV71 infection is associated with significantly increased circulating IL-6, IL-10, and IL-13 in HFMD patients [6]. Moreover, some recent studies have established a role of IL-27 in restricting virus replication via type I IFN [10, 11]. However, the role of IL-27 in EV71 infections is unclear. The purpose of this study was to determine serum IL-27 levels and to evaluate the clinical application of IL-27 as a novel biomarker for early cardiopulmonary failure in EV71-infected children with CNS involvement.

## 2. Materials and Methods

### 2.1. Ethics Statement

The study was approved by the Internal Review and Ethics Boards of Guangdong Medical University, Guangzhou Nansha Center for Disease Control and Prevention, Huashan Hospital, and Guangzhou Nansha Central Hospital, and informed consent was obtained from the parents of each of the enrolled children.

### 2.2. Subjects

 A total of 127 EV71-infected HFMD children admitted to the pediatric department of Guangzhou Nansha Central Hospital between February 2012 and July 2014 were included in the study. The diagnostic criteria of HFMD disease with EV71 infection according to file were as follows: “hand, foot and mouth disease treatment guidelines” published by Health Department of China in 2010 and stool test positive for EV71 virus, as we previously reported [6]. Another 95 healthy children who were EV71-IgM(-) and had no history of HFMD between February 2013 and July 2015 at the Guangzhou Nansha Center for Disease Control and Prevention and Guangzhou Nansha Central Hospital were enrolled as asymptomatic controls. The demographic and clinical characteristics for all study subjects are shown in Table 1. The diagnostic criteria and clinical stage of children with EV71 infections were determined. Children with EV71 HFMD were divided into clinical stage II (characterized by CNS involvement, 55 cases), stage III (characterized by early cardiopulmonary failure, 42 cases), and stage IV (cardiopulmonary failure, 30 cases). According to our previously reported work [6], 10 cases of stage IV patients were followed up, and three time points were settled for serum collection, the day of admission (named TP0), the day the disease improved (named TP1), and the day the disease recovered (named TP2). The criteria for delimiting the day the disease improved were as follows: body temperature dropped under 38°C, CNS involvement was significantly alleviated, and WBC counts and blood glucose level decreased. The criteria for delimiting the day the disease recovered were body temperature, WBC counts, blood glucose, and heart and lung function recovering to normal.

### 2.3. Determination of Serum IL-27 Levels

Serum samples were collected from venous blood at room temperature and stored at −80°C until use. Serum IL-27 levels were measured using the Precoated LEGEND MAX Human IL-27 ELISA (Enzyme Linked Immunosorbent Assay) Kit (BioLegend, San Diego, CA, USA) according to the manufacturer's instructions.

### 2.4. DNA Extraction and SNP Genotyping

Genomic DNA was extracted from peripheral EDTA-blood samples using a TIANamp Blood DNA Kit (Tiangen, Beijing, China) according to the manufacturer's instructions. Three single nucleotide polymorphisms (SNPs), rs153109, rs17855750, and rs181206, in* IL27p28* were selected as candidate sites based on earlier studies demonstrating associations with immune-related diseases [12–18]. Polymerase chain reaction-restriction fragment length polymorphism (PCR–RFLP) was used to detect genotypes. The primer sequences for the rs153109 SNP were F: 5′-TCAGTCAGTGACCAGGATCG-3′ and R: 5′-ACCAAGAAACCCCATCCTCT-3′, the annealing temperature was 58°C, and the PCR products were incubated with PaeR7I restriction enzymes (New England Biolabs, Ipswich, MA, USA) for 4 h. The primer sequences for rs17855750 were F: 5′-ATCTCGCCAGGAAGCTGCGC-3′ and R: 5′-CTGTTAGTGGGGGCCAGAAGGGA-3′, the annealing temperature was 62°C, and the PCR products were incubated with restriction enzymes BstUI restriction enzymes (New England Biolabs) for 8 h. The primer sequences for rs181206 were F: 5′-GCTTCAGCCCTTCCATGCCC-3′ and R: 5′-TCTACCTGGAAGCGGAGGTGCC-3′, the annealing temperature was 64°C, and the PCR products were incubated with FauI restriction enzymes (New England Biolabs) for 12 h. The reactions were performed at 94°C for 1.5 min followed by 34 cycles of 30 s at 94°C, 30 s at the annealing temperature indicated above, 30 s at 72°C, and finally one cycle of 5 min at 72°C. The obtained digestion products were visualized on a 4% agarose gel and stained with Gold View (SBS Genentech, Beijing, China). To validate the method used in this study, PCR-amplified DNA samples from 45 cases (20% of all subjects) randomly selected subjects were examined by DNA sequencing at Invitrogen Biotechnology Company (Guangzhou, China) and the results were 100% concordant.

### 2.5. Statistical Analysis

Statistical analyses were performed as previously described [13, 15, 16] using the GraphPad Prism version 5.0 software (GraphPad Software Inc., San Diego, CA, USA). Comparisons were carried out using Student's *t*-tests or chi-square (*χ* ^2^) tests for 2-group comparisons when appropriate. Correlations were evaluated using Spearman's rank correlation coefficients following our previously described methods [19–21]. To evaluate the quality of the genotyping data, *χ* ^2^ test was used to determine whether SNP genotype frequencies were in Hardy-Weinberg equilibrium. The associations between IL-27 genotypes/alleles and EV71 infection risk were estimated by calculating the odds ratios (OR) and 95% confidence intervals (CI). A *P* value of 0.05 was considered significant.

## 3. Results

### 3.1. Clinical Characteristics for the Study Subjects

The patients were divided into groups corresponding to 3 disease stages, as described previously in the Materials and Methods [6]. No significant difference in age or gender was noted between EV71-infected patients and controls (*P* > 0.05). The frequency of high fever (>39°C for greater than 3 days) at admission, vomiting, skin rash, oral ulcer, being easily frightened, depression, abnormal breathing, increased heart rate, GLU > 8.3 mmol/L, CRT > 2 s, and white blood cell (WBC) counts (×10^9^/L) were higher in EV71-infected patients than in control patients (Table 1).

### 3.2. Serum IL-27 Levels

**Figure 1:**
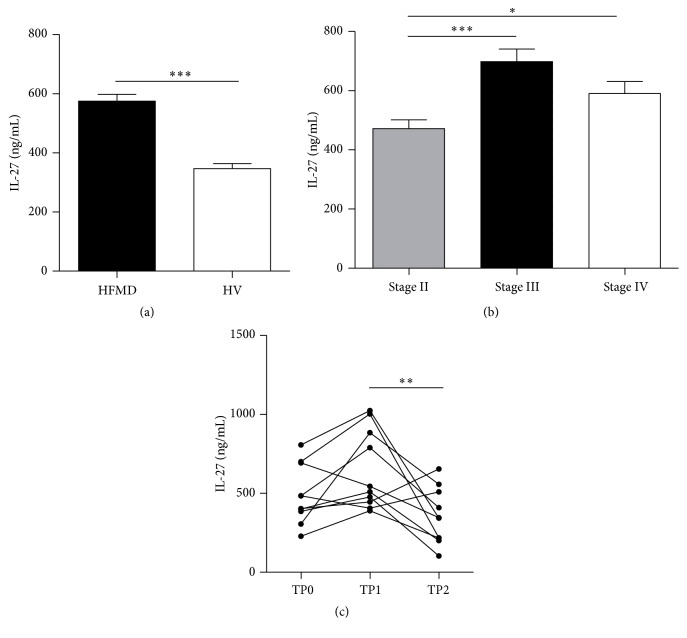
Serum IL-27 levels in EV71-infected patients. Serum IL-27 levels in 127 EV71-infected HFMD patients (including 55 clinical stage II cases, 42 stage III cases, and 30 stage IV cases) and 95 healthy controls were detected by ELISA. Values are expressed as means ± SEM. (a) Serum IL-27 levels were significantly higher in EV71-infected HFMD patients than in healthy controls (*P* < 0.01). (b) Serum IL-27 levels in clinical stage III EV71-infected HFMD patients were higher than clinical stage II and clinical stage IV EV71-infected patients (*P* < 0.05). (c) Serum IL-27 levels in followed-up stage IV patients, the day of admission (TP0), the day the disease improved (TP1), and the day the disease recovered (TP2). ^*∗*^ *P* < 0.05; ^*∗∗*^ *P* < 0.01; ^*∗∗∗*^ *P* < 0.001.

Serum IL-27 levels in 127 EV71-infected HFMD patients (including 55 clinical stage II cases, 42 stage III cases, and 30 stage IV cases) and 95 healthy controls were detected by ELISA. The serum IL-27 levels were significantly higher in EV71-infected HFMD patients than in healthy controls (*P* < 0.01), as shown in Figure 1(a). We also found that serum IL-27 levels were distinctly higher in clinical stage III EV71-infected HFMD patients than in clinical stage II or clinical stage IV EV71-infected patients (Figure 1(b)). Interestingly, the serum IL-27 levels increased after disease improved and significantly decreased after disease recovered in followed-up stage IV patients (Figure 1(c)). These results suggested that IL-27 may play a role in HFMD caused by EV71 infection, especially in patients with early cardiopulmonary failure, a major characteristic of clinical stage III EV71-infected HFMD patients.

### 3.3. Relationship between Serum IL-27 Levels and C-Reaction Protein (CRP) Levels

**Figure 2:**
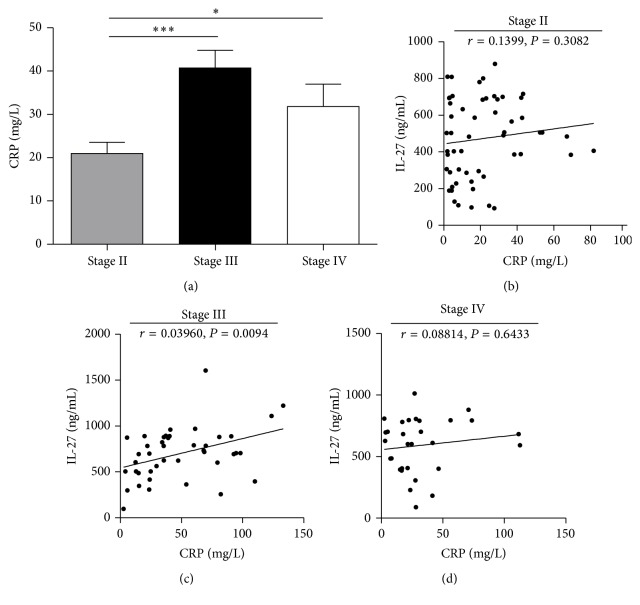
Correlation of serum IL-27 levels with CRP levels. (a) CRP levels in stage II, III, and IV EV71-infected patients. (b) Correlations of serum IL-27 levels with CRP levels in stage II EV71-infected patients. (c) Correlations of serum IL-27 levels with CRP levels in stage III EV71-infected patients. (d) Correlations of serum IL-27 levels with CRP levels in stage IV EV71-infected patients. ^*∗*^ *P* < 0.05; ^*∗∗∗*^ *P* < 0.001.

Consistent with previous studies [22, 23], we found that blood CRP levels were related to the severity of EV71-infected HFMD, as shown in Figure 2(a). And there was a marked correlation between CRP levels and IL-27 levels in clinical stage III EV71-infected HFMD patients (Spearman's *r* = 0.3960, *P* = 0.0094) but not in clinical stage II or IV EV71-infected HFMD patients as shown in Figures 2(b)–2(d). These results suggested that aberrant expression of IL-27 may associate with CRP to mediate disease process in clinical stage III EV71-infected HFMD patients.

### 3.4. Relationship between Serum IL-27 Levels and* IL27p28* Polymorphism

**Figure 3:**
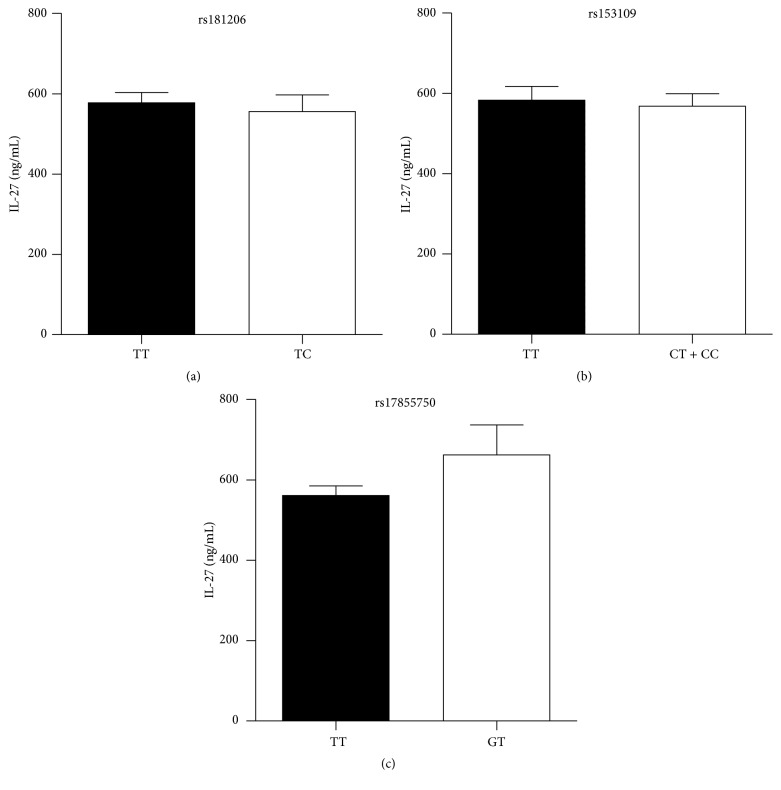
Relationship between serum IL-27 levels and* IL27p28* polymorphism in EV71-infected patients. Serum IL-27 levels were measured in 127 individuals for each* IL27p28* polymorphism (rs181206, rs153109, and rs17855750) in EV71-infected patients. Values are expressed as means ± SEM. No associations were found between rs181206 (a), rs153109 (b), and rs17855750 (c) and serum IL-27 levels in EV71-infected patients.

In order to further revealing the influence of* IL27p28* polymorphism on serum IL-27 levels, the genotype and allele frequencies of the rs153109, rs17855750, and rs181206 SNPs in* IL27p28* in EV71-infected patients were detected, and the results are shown in Table 2. The genotype distributions of the three SNPs in the EV71-infected patients and the controls were in Hardy-Weinberg equilibrium (*P* > 0.05). However, the genotype and allele frequencies were not associated with EV71 infection risk (*P* > 0.05; Table 2) or clinical stage in EV71-infected patients (*P* > 0.05; Table SI in Supplementary Material available online at http://dx.doi.org/10.1155/2016/4025167). To determine the relationship between serum IL-27 levels and* IL27p28* polymorphisms, serum IL-27 levels were estimated for each genotype at the three polymorphic sites. No associations were detected between* IL27p28* polymorphisms (rs153109, rs17855750, and rs181206) and serum IL-27 levels in EV71-infected patients (*P* > 0.05), as shown in Figure 3.

## 4. Discussion

The current work extends our previous studies of IL-27 functions in EV71-infected HFMD patients with CNS involvement and demonstrates the following previously undescribed findings. (i) A dynamic change of blood IL-27 levels was observed on disease severity from stage II to stage IV, and IL-27 may be a novel prediction biomarker for early cardiopulmonary failure in EV71-infected children with CNS involvement. (ii) Increased serum IL-27 levels are related to blood CRP levels in stage III patients with early cardiopulmonary failure. (iii)* IL27p28* polymorphisms (rs153109, rs17855750, and rs181206) were not associated with blood IL-27 levels, EV71 infection risk, or clinical stage.

IL-27 functions as an early mediator of innate and adaptive immune responses. It promotes the generation and effector functions of antigen-specific CD8^+^ T cells [24–26], modulates Foxp3-expressing regulatory T cell responses [27], and programs effector T cells into a unique T-effector stem cell phenotype in the tumor microenvironment [28]. It also induces molecular pathways that are involved in the recruitment and activation of natural killer cells and natural killer T cells [28–31]. In addition, IL-27 inhibits cytotoxic T lymphocyte- (CTL-) mediated platelet destruction in primary immune thrombocytopenia [32].

Recently, the passive and active roles of IL-27 in the pathogenesis of several viral diseases have been closely examined. IL-27 has an active role in the suppression of proinflammatory cytokine-associated liver toxicity via the inhibition of IL-12 and IFN-*γ* production [33] and inhibits HIV replication in peripheral blood mononuclear cells and macrophages [34]. IL-27 also inhibits CNS autoimmunity by inhibiting the polarization of human T cells to the Th1 and Th17 effector pathways [35] and impairs the control of CNS virus replication via the induction of IL-10 in virus-specific CD4^+^ T cells [9]. Therefore, we hypothesized that IL-27-mediated immune responses may be related to EV71 infections, especially severe infections.

Accordingly, we investigated blood IL-27 levels with disease severity and EV71 virus loads. We found that serum IL-27 levels were higher in EV71-infected HFMD patients than in healthy controls. We also observed a rapid increase of serum IL-27 levels in clinical stage III EV71-infected HFMD patients, in the process, serum IL-27 levels marked correlation with CRP levels, suggesting IL-27 may be a novel prediction biomarker for early cardiopulmonary failure in EV71-infected children with CNS involvement. It is worth noting that no associations were detected between IL-27 levels and virus loads (data not shown). The results suggested that IL-27 may not mediate the control of virus replication in EV71-infected patients, different from HIV-infected patients or JHMV-induced encephalomyelitis.

The associations between* IL27p28* polymorphisms and the risk of various diseases such as asthma [13–18, 36–43], chronic obstructive pulmonary disease [13–18, 37–43], coronary heart disease [13–18, 38–43], rheumatoid arthritis [14–18, 38–43], inflammatory bowel disease [14–18, 39–43], pulmonary tuberculosis [15–18, 40–43], allergic rhinitis [15–18, 41–43], HCV infection [16–18, 41–43], and several tumors (e.g., esophageal cancer [16–18, 42, 43], nasopharyngeal carcinoma [16, 17, 42, 43], ovarian [16, 17], and bladder [16] cancer) have also been detected. Therefore,* IL27p28* polymorphisms have been investigated in this study. Our data suggested that the* IL27p28* SNPs rs153109, rs17855750, and s181206 are not associated with EV71 infection risk or clinical stage. We also did not detect associations between serum IL-27 levels and* IL27p28* gene polymorphisms (rs153109, rs17855750, and rs181206), similar to the results of Zhao et al. [12] and Tang et al. [44]. These results suggest that the three SNPs do not influence IL-27 production. Interestingly, we found that IL-27 may be related to early cardiopulmonary failure, a major characteristic of clinical stage III EV71-infected HFMD patients. Consistent with previous reports, we also found that blood CRP levels increased with the severity of HFMD disease [22, 23] and the increased CRP levels positive correlation with IL-27 levels in stage III patients with early cardiopulmonary failure. Taken together, our results indicate that patients in clinical stage III typically produce high levels of IL-27; IL-27 may be a novel prediction biomarker at the acute stage in EV71-infected children with CNS involvement.

This finding may be related to the strong proinflammatory reaction that is regulated by the host immune system against EV71 infection. IL-27 can be used as an indicator of disease severity and a marker for prognosis for EV71 infection.

## Supplementary Material

The Supplementary Material is the Genotype and allele frequencies of IL27*p28* polymorphism in EV71-infected patients.

## Figures and Tables

**Table 1:** Summary of clinical data.

Groups	EV71-infected patients			Controls (*n* = 95)
	Stage II (*n* = 55)	Stage III (*n* = 42)	Stage IV (*n* = 30)	
Female/male	27/28	20/22	16/14	48/47
Age (months), mean ± SD	25.31 ± 10.24	26.55 ± 11.23	26.21 ± 10.47	26.11 ± 11.05
High fever (>39°C >3 days at admission), *n* (%)	33 (60.00)	25 (59.52)	20 (66.67)	0 (0)
Vomiting, *n* (%)	15 (27.27)	13 (30.95)	13 (43.33)	0 (0)
Skin rash, *n* (%)	52 (94.55)	40 (95.24)	29 (96.67)	0 (0)
Oral ulcer, *n* (%)	53 (96.36)	42 (100)	29 (96.67)	0 (0)
Easily frightened, *n* (%)	8 (14.55)	10 (23.81)	12 (40.00)	0 (0)
Depression, *n* (%)	16 (29.09)	18 (42.86)	25 (83.33)	0 (0)
Abnormal breathing, *n* (%)	9 (16.36)	8 (19.05)	15 (50.00)	0 (0)
Increased heart rate, *n* (%)	25 (45.45)	26 (61.90)	26 (86.67)	0 (0)
GLU > 8.3 mmol/L, *n* (%)	30 (54.55)	30 (71.43)	25 (83.33)	0 (0)
CRT > 2 s, *n* (%)	8 (14.55)	25 (59.52)	23 (76.67)	0 (0)
WBC (×10^9^/L), mean ± SD	7.08 ± 3.41	8.74 ± 3.33	11.50 ± 3.37	6.78 ± 2.27

WBC: white blood cell.

**Table 2:** Genotype and allele frequencies of *IL27p28* polymorphism in EV71-infected patients and controls.

SNP	Genotype and allele	EV71-infected patients (*n* = 127), *n* (%)	Controls (*n* = 95), *n* (%)	*χ* ^2^	*P* values	Unadjusted OR (95% CI)
rs153109	TT	57 (44.88)	42 (44.21)	0.010	0.921	1.028 (0.602–1.755)
	CT	56 (44.09)	43 (45.26)	0.030	0.862	0.9538 (0.559–1.628)
	CC	14 (11.02)	10 (10.53)	0.014	0.906	1.053 (0.446–2.486)
	T	170 (66.93)	127 (66.84)	0.000	0.985	1.004 (0.673–1.497)
	C	84 (33.07)	63 (33.16)	0.000	0.985	0.996 (0.668–1.485)

rs17855750	TT	110 (86.61)	85 (89.47)	0.416	0.519	0.761 (0.332–1.748)
	GT	17 (13.39)	10 (10.53)	0.416	0.519	1.314 (0.572–3.016)
	T	237 (93.31)	180 (94.74)	0.389	0.533	0.775 (0.346–1.732)
	G	17 (6.69)	10 (5.26)	0.389	0.533	1.291 (0.557–2.888)

rs181206	TT	111 (87.40)	83 (87.37)	0.000	0.994	1.003 (0.450–2.234)
	TC	16 (12.60)	12 (12.63)	0.000	0.994	0.997 (0.448–2.221)
	T	238 (93.70)	178 (93.68)	0.000	0.994	1.003 (0.463–2.173)
	C	16 (6.30)	12 (6.32)	0.000	0.994	0.997 (0.460–2.161)
