# Supplementary material for: Interleukin-27 as a Novel Biomarker for Early Cardiopulmonary Failure in Enterovirus 71-Infected Children with Central Nervous System Involvement
Source: Mediators Inflamm. 2016 Jun 15;2016:4025167. doi: 10.1155/2016/4025167 (PMC4925946; doi:10.1155/2016/4025167)
Supplement: Supplementary file 1 — The Supplementary Material is the Genotype and allele frequencies of IL27p28 polymorphism in EV71-infected patients. [file 4025167.f1.pdf]

Supplementary Digital Content 1

TABLES SI. Genotype and allele frequencies of IL-27 *p28* polymorphism in EV71-infected patients

SNP	Genotype and allele	Stage II (n = 55)	Stage III-IV (n = 72)	χ^2	*P*-value	Unadjusted OR (95% CI)
rs153109						
	TT	24 (43.64)	33 (45.83)	0.061	0.805	0.915 (0.451–1.854)
	CT	25 (45.45)	31 (43.06)	0.073	0.787	1.102 (0.544–2.235)
	CC	7 (12.73)	7 (9.72)	2.027	0.155	2.479 (0.687–8.945)
	T	73 (66.36)	97 (67.36)	0.028	0.867	0.956 (0.564–1.620)
	C	39 (35.45)	45 (31.25)	0.498	0.480	1.208 (0.714–2.045)
rs17855750						
	TT	47 (85.45)	63 (87.50)	0.113	0.737	0.839 (0.301–2.339)
	GT	8 (14.55)	9 (12.5)	0.113	0.737	1.191 (0.428–3.320)
	T	102 (92.73)	135 (93.75)	0.105	0.747	0.850 (0.317–2.280)
	G	8 (7.27)	9 (6.25)	0.105	0.747	1.176 (0.439–3.156)
rs181206						
	TT	48 (87.27)	63 (87.50)	0.001	0.970	0.980 (0.340–2.819)
	TC	7 (12.73)	9 (12.5)	0.001	0.970	1.021 (0.355–2.938)
	T	103 (93.64)	135 (93.75)	0.001	0.971	0.981 (0.354–2.722)
	C	7 (6.36)	9 (6.25)	0.001	0.971	1.019 (0.367–2.829)
